# The current status of phlebotomine sand flies in Albania and incrimination of *Phlebotomus neglectus* (Diptera, Psychodidae) as the main vector of *Leishmania infantum*

**DOI:** 10.1371/journal.pone.0179118

**Published:** 2017-06-19

**Authors:** Enkelejda Velo, Gioia Bongiorno, Perparim Kadriaj, Teita Myrseli, James Crilly, Aldin Lika, Kujtim Mersini, Trentina Di Muccio, Silvia Bino, Marina Gramiccia, Luigi Gradoni, Michele Maroli

**Affiliations:** 1 Department for Control of Infectious Diseases, Institute of Public Health, Tirana, Albania; 2 Unit of Vector-borne Diseases and International Health, MIPI Department, Istituto Superiore di Sanità, Rome, Italy; 3 Department of Animal Health, Food Safety and Veterinary Institute, Tirana, Albania; 4 Independent Researcher, Rome, Italy; Academic Medical Centre, NETHERLANDS

## Abstract

The incidence of visceral leishmaniasis (VL) in Albania is higher than in other countries of southern Europe, however the role of local sand fly species in the transmission of *Leishmania infantum* was not addressed conclusively. In 2006, a country-wide collection of sand flies performed in 14 sites selected based on recent occurrence of VL cases showed that *Phlebotomus neglectus* was by far the most prevalent species (95.6%). Furthermore, 15% of pools made from 422 *P*. *neglectus* females tested positive for *Leishmania* sp. genomic DNA. In the same year, *Culicoides* trapping was performed for bluetongue disease surveillance in 91 sites of southern Albania, targeting livestock farms regardless recent occurrence of VL in the surveyed areas. In 35 sites where sand flies were collected along with midges, *Phlebotomus perfiliewi* was the most prevalent among the *Phlebotomus* species identified, however search for leishmanial DNA in females of this species was unsuccessful. In 2011, sand flies were trapped in 4 sites of north Albania characterized by high VL incidence, and females were dissected to search for *Leishmania* infections. Both *P*. *neglectus* and *P*. *tobbi* were collected at high densities. Two positive specimens were detected from a sample of 64 *P*. *neglectus* trapped in one site (3.1%). Parasites were successfully cultured from one specimen and characterized as belonging to *Leishmania infantum* zymodeme MON-1, the only zymodeme so far identified as the agent of human and canine leishmaniasis in the country. Altogether our studies indicate that *P*. *neglectus* is the main leishmaniasis vector in Albania.

## Introduction

Among the Mediterranean countries endemic for visceral leishmaniasis (VL), Albania is recorded as one of the most affected [1]. Unlike the other South West European countries, the disease occurs predominantly in children [2] although cases of HIV/*Leishmania* co-infection are recently increasing in adults. VL cases occur in urban settlements on the Adriatic coastal plain and the adjoining river valleys. About 80% of patients notified to the Institute of Public Health from 1995 through 2014 were from peripheral districts of Shkoder and Lezhë in the north, Tirana in the centre and Lushnjë, Fier, Berat and Vlorë in the south. The remaining cases were from rural areas in coastal or lake territories. It is likely that a proportion of VL cases occurred in remote rural areas are left undiagnosed or unreported. Although very underreported, cutaneous leishmaniasis (CL) is seen with higher rates from Lezhë, Elbasan, Krujë, Tiranë and Berat districts (data recorded by the Institute of Public Health, Tirana).

Since late 1980s, a number of parasite isolates from human (VL and CL) and canine leishmaniasis cases were obtained and identified biochemically or molecularly as *Leishmania infantum*. Some of them, typed by Multilocus Enzyme Electrophoresis (MLEE), were found to belong to zymodeme Montpellier (MON)-1, the commonest zymodeme agent of VL in the Mediterranean region (Table 1).

**Table 1:** Albanian *Leishmania* isolates identified from 1988 through 2012 by MultiLocus Enzyme Electrophoresis (MLEE) or PCR-restriction fragments length polymorphism of ribosomal internal transcribed spacer-1 (ITS-1 PCR-RFLP).

Strain/Sample Code	Year	Host	Clinical form	Place, District	Typing method	*Leishmania* identification
**MCAN/AL/1988/ISS429**	1988	Dog	Canine leishmaniasis	Tirane	MLEE	*L*. *infantum* MON-1
**MCAN/AL/1998/C78L574-ISS1761**	1998	Dog	Canine leishmaniasis	Tirane	MLEE	*L*. *infantum* MON-1
**MHOM/AL/2006/ISS2840-ZR**	2006	Human	Visceral leishmaniasis	Patos, Fier	MLEE	*L*. *infantum* MON-1
**MCAN/AL/2006/ISS2841**	2006	Dog	Canine leishmaniasis	Tirane	MLEE	*L*. *infantum* MON-1
**MHOM/AL/2007/ISS2849-FT**	2007	Human	Visceral leishmaniasis	Lushnje	ITS-1 PCR-RFLP	*L*. *infantum*
**MHOM/AL/2007/ISS2850-KH**	2007	Human	Visceral leishmaniasis	Devoll	ITS-1 PCR-RFLP	*L*. *infantum*
**MHOM/AL/2010/ISS2996-ED**	2010	Human	Visceral leishmaniasis	Burrel, Mat	ITS-1 PCR-RFLP	*L*. *infantum*
**RK2012-CL**	2012	Human	Cutaneous leishmaniasis	Shijak, Durres	ITS-1 PCR-RFLP	*L*. *infantum*

Robust knowledge of the domestic/peridomestic phlebotomine sand fly fauna was acquired through several entomological surveys performed all over Albania during the 1958–1989 period (reviewed by Adhami and Murati [3]). Results from these investigations showed the endemic presence of three members of the *Phlebotomus* (*Larroussius*) subgenus, namely *Ph*. *neglectus*, *Ph*. *perfiliewi* and *Ph*. *tobbi*, all species incriminated as *L*. *infantum* vectors elsewhere in the Mediterranean [4–6]. Some years later (2002), a study on the sand fly fauna in two areas of central (Kruje district) and northern Albania (Lezhë district) confirmed the presence of the above *Larroussius* species, among which *P*. *neglectus* was found predominant (75.6%) [7]. In this study, natural *Leishmania* infections where searched by molecular techniques in a small number of *P*. *neglectus* females, which were found negative. Ultimately, the role of *Larroussius* phlebotomine members in the transmission of *L*. *infantum* in Albania was not substantially addressed so far. In this paper, we report the results of three entomological surveys carried out in several leishmaniasis endemic areas and provide evidence for the definitive incrimination of *Ph*. *neglectus* as the main VL vector in Albania.

## Materials and methods

### Periods of sand fly collection and description of sites

**Fig 1:**
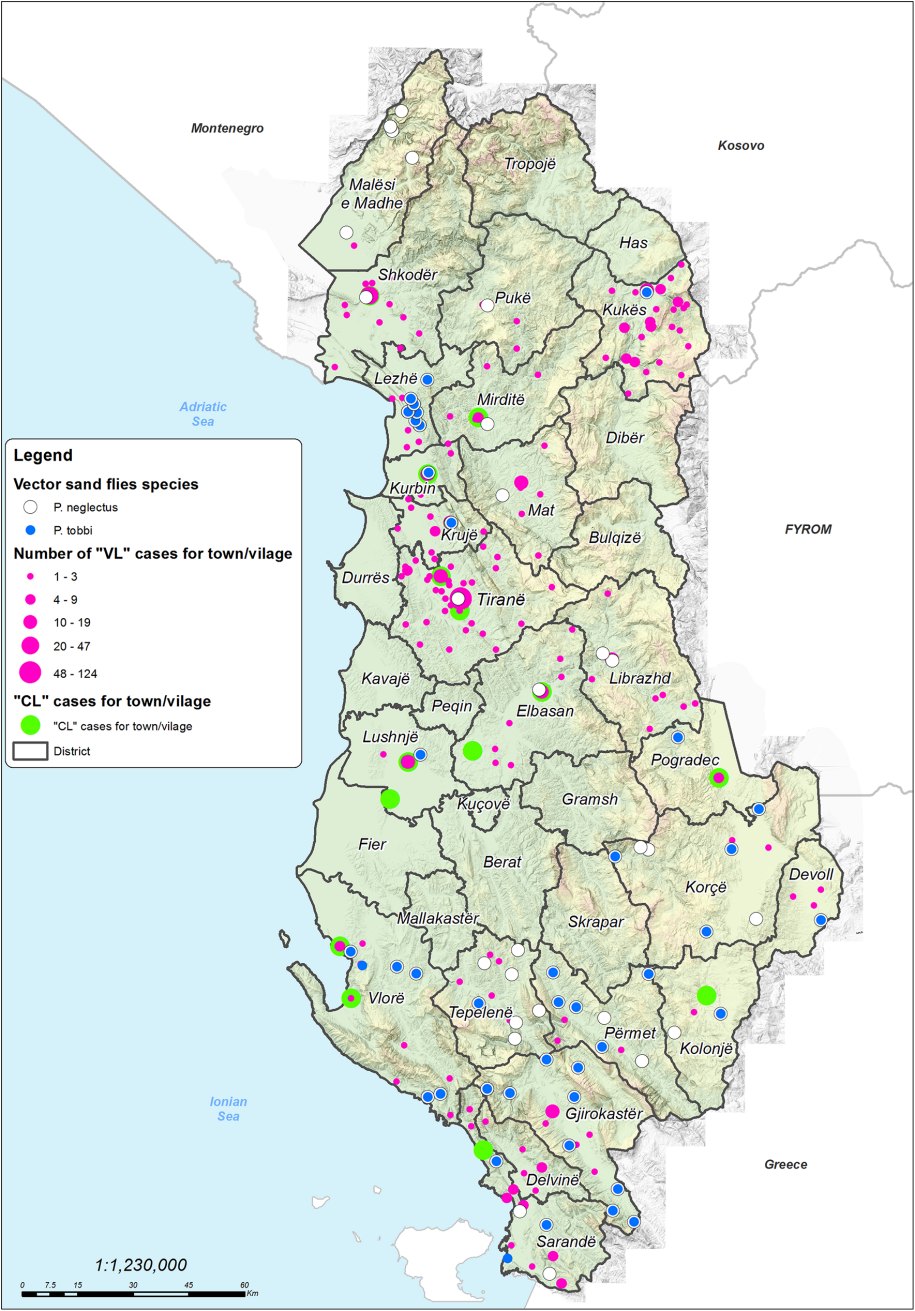
Spatial distribution of sand fly sampling sites and human leishmaniasis cases in the study area for the period 2001–2014.

**Fig 2:**
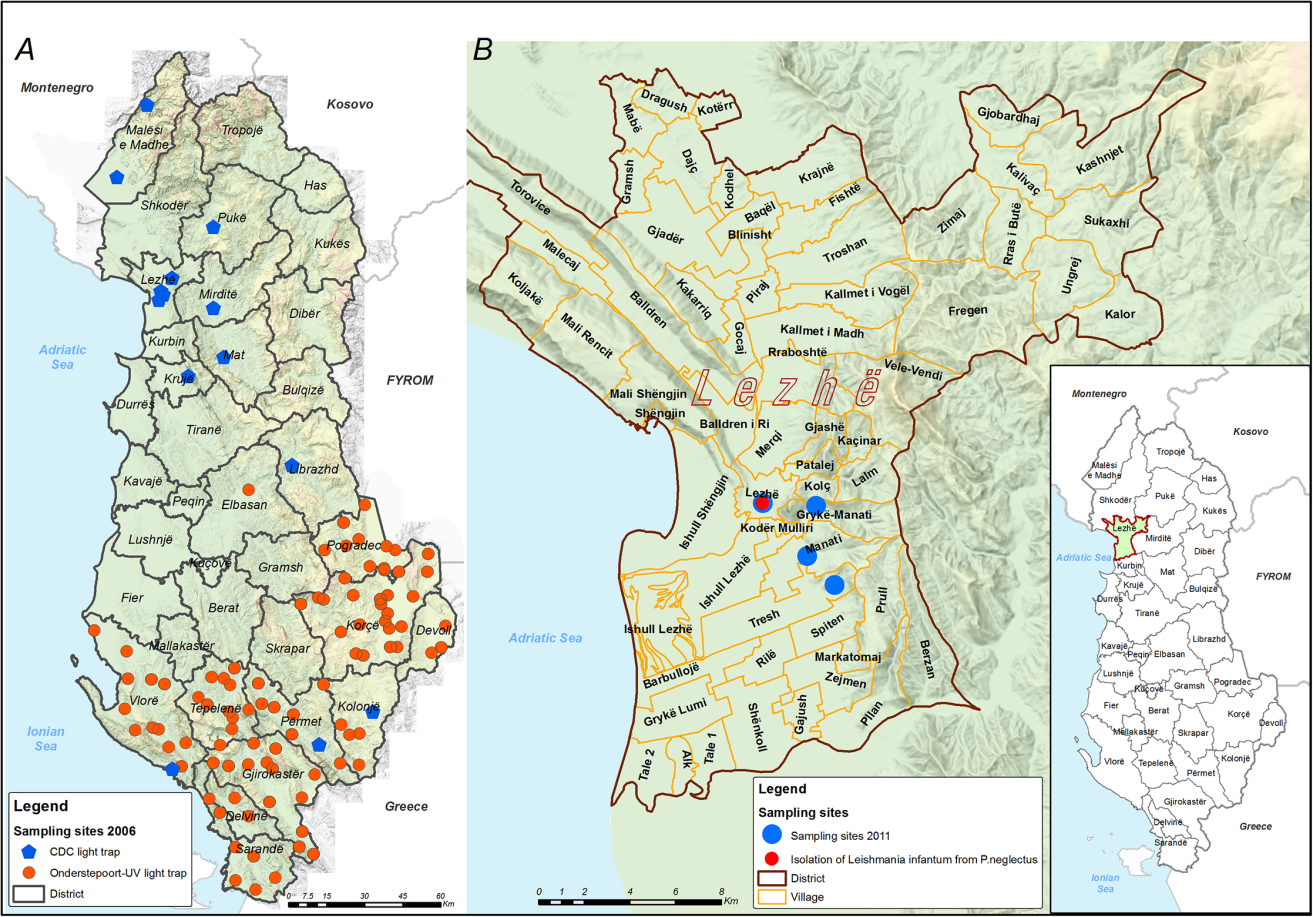
**Sand fly collection sites (A and B).** Map showing: (A) 2006 sampling sites targeting foci of recent VL transmission and *Culicoides* midges monitoring in the frame of a bluetongue-disease surveillance program (see text for the sampling frame design); (B) 2011 sampling sites targeting foci of recent VL transmission.

In total, 132 sites were investigated for sand flies during 2001–2014 (Fig 1), of which 109 across three studies (Fig 2). No specific permissions were required for these locations/activities. Landowners gave permission to conduct the studies on their properties. The field studies did not involve endangered or protected species.

#### Study 1. Country-wide collections targeting VL endemic sites

Between June and September 2006 sand flies were collected in 10/36 districts, representative of northern (4), central (3) and southern territories (3), with the aim of providing further information on sand fly species distribution and prevalence, and to search for *Leishmania* infections in putative vectors by means of molecular methods. The survey included a total of 14 sites. Target areas consisted of small villages or peri-urban quarters of towns where VL cases were recently reported in resident population. Sites consisted of cow barns and chicken pens near houses. A census of potential sand fly hosts revealed communities made of humans (4–15), dogs (1–4), cats (1–6), cows (1–4), sheep (0–40), pigs (0–10), chickens (5–15) and rabbits (0–8). Altitude of sites varied between the districts, ranging 5–499 m above sea level (a.s.l.) for most of them (8 districts), and 806–1000 m for 2 mountainous districts.

#### Study 2. Sites monitored in three southern districts

Between May and October 2006 sand flies were collected by taking advantage of a systematic *Culicoides* midges trapping in the frame a bluetongue disease surveillance program. A total of 91 sites in 9 counties were investigated, located in three southernmost districts of Vlorë, Gjirokastër and Korçë. Target sites were farms with presence of pigs, equines or ruminant livestock. A sampling frame based on 88 km^2^ sections was applied due to the cross-sectional nature of the study and the wide range of habitats present over a small geographical territory. Using Arcview9 GIS software (ESRI, 2005), the sampling frame was superimposed on a map layer of the study area. A code was given to each section in order to identify that specific area and the closest village to the middle of the section was used as the sampling point. Altitudes of sites varied between the three districs, ranging from 2–702 m a.s.l. in Vlorë, 149–977 m a.s.l. in Gjirokastër and 517–1322 m a.s.l. in Korçë. Farms chosen as trapping sites satisfied specific criteria including the presence of at least 5 livestock units (e.g. cattle, goats, sheep, pigs, horses or donkeys), location at least 2.5 km from the sea coast, and no insecticides used during the past 6 months.

#### Study 3. Sites with recent high VL incidence

A survey designed for *Leishmania* isolation and characterization from dissected sand fly specimens, was performed in September 2011 in rural and peri-urban sites of 4 villages of Lezhë district. The choice of representative sites for sand fly collections was based on the following: i) high cumulative incidence of VL in the population; ii) previous knowledge (from Study 1) of the endemic presence of potential *L*. *infantum* vector species, and iii) logistic considerations, such as a convenient distance from the equipped laboratory necessary for microscopy dissections and cultures to be performed in aseptic conditions.

### Sand fly collection and identification

Catches were performed using two methods, standard CDC miniature light traps (Hausherr’s Machine Works, Toms River, NJ, USA) worldwide used for sand flies collection, and Onderstepoort-type blacklight traps with 8 W UV-light bulbs and downdraught suction motors which have *Culicoides* as main target species. At best of our knowledge, no studies are available on the comparative effectiveness between these two types of traps for the collection of sand flies. In Studies 1 and 3, two-three CDC traps per site were set in protected habitats such as inside or close to animal pens, in courtyards adjacent to houses or under the eaves of buildings. Traps were suspended at approximately 1.5 meters above the ground and were operated from one hour before sunset until one hour after sunrise. The light traps were retrieved each morning and the collected insects taken alive to laboratory for processing.

In Study 2, catches were carried out using 10 Onderstepoort-type traps for *Culicoides* monitoring. In each collecting site, one trap was used for one night only. The traps were positioned outdoor within 25 m from the housed livestock, 1.5–2 m above ground, and were operated overnight. The resultant insect catch was poured through a fine gauze square and then transferred to a plastic jar containing 70% alcohol for transportation.

In both types of catches, phlebotomines were sorted under a binocular microscope, counted and stored in alcohol prior to their identification following standard taxonomic keys [8,9].

### Examination of sand fly specimens for *Leishmania* infection

#### Detection of *Leishmania* genomic DNA

Nested (n)-PCR was used to detect *Leishmania* genomic DNA in groups of females pooled by species, site and date of collection. The specimens were homogenized in 1.5 ml sterile tubes using a plastic pestle. 40 μL lysis buffer (100 mM TRIS-HCI, 100 mM NaCI, 25 mM EDTA, 0.5% SDS, pH 8) was added and the homogenate was digested overnight at 37°C by 2 μg/μL proteinase K (Promega). Genomic DNA was extracted by phenol-chloroform and precipitated with 100% ethanol and then centrifuged for 30 min at 13,000g. The DNA pellet was resuspended in 50 μL of sterile water and stored at—20°C until use. Small-subunit ribosomal DNA was amplified by n-PCR technique by sequential amplification of gene fragments using kinetoplastid specific primers R221 and R332, and *Leishmania* sp. primers R223 and R333 [10]. The cycling conditions were denaturation at 94°C for 30 s, annealing at 60°C for 30 s (65°C for 30 s for the second PCR) and extension at 72°C. Two negative controls (DNA from uninfected flies and no DNA) and two positive controls (*L*. *infantum* DNA and *Phlebotomus* plus *L*. *infantum* DNA) were used in all experiments. Finally, the amplification products were analysed through a 1.5% agarose gel and visualized under UV light. Positive samples were anticipated to yield a predicted n-PCR product of 358 bp.

#### Isolation of *Leishmania* parasites

Before dissection the flies were anaesthetized for 5 min in a deep freezer and stored in sterile phosphate-buffered saline containing gentamicin (250 g/ml), 5-fluorocytosine (500 g/ml) and commercial baby shampoo (1 drop/30 ml). The specimens were identified by the morphology of pharyngeal armature and spermathecae according to Léger et al [9]. When flies were found to harbor promastigotes, some drops of Evans' Modified Tobie's Medium (EMTM) liquid phase were added to the dissected material and then the entire gut was aspirated and inoculated into screw-top vials containing EMTM solid phase [11].

#### Characterization of *Leishmania* isolates

*Leishmania* typing was performed by both molecular and biochemical methods. PCR-RFLP analysis used primers LITSR and L.5.8S amplifying the internal transcribed spacer-1 (ITS-1) sequence separating the genes coding for SSU rRNA and 5.8S rRNA [12,13]. *Leishmania* DNA from the WHO reference strain for *L*. *infantum* (MHOM/TN/1980/IPT-1) and from two Albanian *L*. *infantum* strains of human and canine origin (MHOM/AL/2006/ISS2840 and MCAN/AL/2006/ISS2841, respectively), were used as reference. Ten μl of PCR products were digested overnight in a total volume of 20 μl, with 10U of *Hae*III restriction enzyme, as recommended by the manufacturer (Promega). PCR-RFLP products were subjected to electrophoresis by 4% MethaPhor gel (EuroClone) or by Qiaxcel capillary electrophoresis (Qiagen GmbH, Hilden, Germany).

MLEE was employed for the analysis of 15 enzymatic systems following the Montpellier (MON) methodology and zymodeme nomenclature [14]: phosphoglucomutase (PGM; E.C.2.7.5.1); glucose-phosphate isomerase (GPI; E.C.5.3.1.9); glutamate-oxaloacetate transaminases (GOT1, GOT2; E.C.2.6.1.1.); malic enzyme (ME; E.C.1.1.1.40); phosphogluconate dehydrogenase (6PGD; E.C.1.1.1.44); glucose-6-phosphate dehydrogenase (G6PD; E.C.1.1.1.49); malate dehydrogenase (MDH; E.C.1.1.1.37); nucleoside phosphorylases 1 and 2 (NP1, NP2; E.C.2.4.2.1, E.C.4.2.1.*); mannose-phosphate isomerase (MPI; E.C.5.3.1.8); isocitrate dehydrogenase (ICD; E.C.1.1.1.42); diaphorase NADH (DIA; E.C.1.6.2.2); glutamate-dehydrogenase (GLUD; E.C.1.4.1.3); fumarate hydratase (FH; E.C.4.2.1.2) [14]. *L*.*infantum* MON-1 (MHOM/TN/80/IPT-1), *L*.*major* MON-4 (MHOM/SU/73/5-ASKH) and *L*.*tropica* MON-60 (MHOM/SU/74/K27) were used as reference.

## Results

### Sand fly monitoring

#### Sand fly species identified and relative abundance

A total of 1,666 sand fly specimens (28.5% males) was collected in the 3 studies (Table 2).

**Table 2:** Sand fly species diversity and cumulative relative abundance recorded during three entomological studies carried out in Albania in 2006 and 2011.

Study year	Specimens(M%)	Species (%)					
		*P*. *neglectus*	*P*. *tobbi*	*P*. *perfiliewi*	*P*. *papatasi*	*P*. *similis*	*S*. *minuta*
2006^a^	549 (28.3)	525	5	1	0	0	18
2006^b^	730 (43.7)	100	52	213	0	14	351
2011^c^	387 (NR)	216	145	0	5	0	21
Total	1666 (28.5)	841 (50.5)	202 (12.2)	214 (12.8)	5 (0.3)	14 (0.8)	390 (23.4)

^a^ VL-targeted country-wide survey (Study 1)

^b^ Bluetongue-disease surveillance in south Albania, (Study 2)

^c^ Study aimed at *Leishmania* identification from dissected sand flies in sites of elevated VL transmission (Study 3); M: male; NR: species unrecorded in males.

They included 5 *Phlebotomu*s species belonging to three subgenera (*Larroussius*, *Phlebotomus* and *Paraphlebotomus*) and one species of *Sergentomyia*. *Ph*. *neglectus* was the most abundant species (50.5% among all phlebotomines; 65.9% among *Phlebotomus* species) followed by other *Larroussius* species, *Ph*. *perfiliewi* (12.8%/16.8%) and *Ph*. *tobbi* (12.2%/15.8%). A few specimens of *Ph*. *similis* and *Ph*. *papatasi* were also collected, whereas *S*. *minuta* represented 23.4% of all phlebotomine specimens.

#### Spatial distribution

Tables 3 and 4 show the relative abundance by county and district of the sand fly species recorded in Studies 1 and 2, respectively.

**Table 3:** Prevalence and sand fly species identified by county and district in the 2006 country-wide study targeting VL endemic sites (Study 1).

Prefecture	District	Specimens (M%)	Species (%)			
			*P*. *neglectus*	*P*. *tobbi*	*P*. *perfiliewi*	*S*. *minuta*
Vlorë	Vlorë	26 (7.7)	7	0	1	18
	*Total (%)*	26 (7.7)	7 (26.9)	0 (0.0)	1 (3.8)	18 (69.3)
Durrës	Krujë	31 (0.0)	31	0	0	0
	*Total (%)*	31 (0.0)	31 (100.0)	0 (0.0)	0 (0.0)	0 (0.0)
Gjirokastër	Përmet	22 (36.4)	17	5	0	0
	*Total (%)*	22 (36.4)	17 (77.3)	5 (22.7)	0 (0.0)	0 (0.0)
Korçë	Kolonjë	40 (7.5)	40	0	0	0
	*Total (%)*	40 (7.5)	(100.0)	0 (0.0)	0 (0.0)	0 (0.0)
Lezhë	Lezhë	130 (19.2)	130	0	0	0
	Mirditë	1 (100.0)	1	0	0	0
	*Total (%)*	131 (19.8)	131 (100.0)	0 (0.0)	0 (0.0)	0 (0.0)
Elbasan	Librazhd	2 (100.0)	2	0	0	0
	*Total (%)*	2 (100.0)	2 (100.0)	0 (0.0)	0 (0.0)	0 (0.0)
Shkodër	Malësi e Madhe	220 (37.7)	220	0	0	0
	Pukë	65 (52.3)	65	0	0	0
	*Total (%)*	285 (41.4)	285 (100.0)	0 (0.0)	0 (0.0)	0 (0.0)
Dibër	Mat	12 (0.0)	12	0	0	0
	*Total (%)*	12 (0.0)	12 (100.0)	0 (0.0)	0 (0.0)	0 (0.0)
**Overtotal**		**549 (28.3)**	**525 (95.6)**	**5 (0.9)**	**1 (0.2)**	**18 (3.3)**

M = male

**Table 4:** Prevalence and sand fly species by county and district collected in the 2006 *Culicoides*-monitoring study in southernmost counties of Albania (Study 2).

Prefecture	District	Specimens (M%)	Species (%)				
			*P*. *neglectus*	*P*. *tobbi*	*P*. *perfiliewi*	*P*. *similis*	*S*. *minuta*
Vlorë	Delvinë	23 (0.0)	0	0	0	0	23
	Sarandë	258 (34.5)	24	6	56	0	172
	Vlorë	144 (82.1)	4	15	116	0	9
	*Total (%)*	425 (47.1)	28 (6.6)	21 (4.9)	172 (40.5)	0	204 (48.0)
Gjirokastër	Gjirokastër	187 (31.5)	46	21	14	14	92
	Përmet	61 (27.9)	7	5	12	0	37
	Tepelenë	27 (88.9)	13	3	9	0	2
	*Total (%)*	275 (40.4)	66 (24.0)	29 (10.5)	35 (12.8)	14 (5.1)	131 (47.6)
Korçë	Devoll	2 (0.0)	0	0	1	0	1
	Korçë	26 (30.7)	6	2	4	0	14
	Pogradec	2 (0.0)	0	0	1	0	1
	*Total (%)*	30 (26.7)	6 (20.0)	2 (6.7)	6 (20.0)	0 (0.0)	16 (53.3)
**Overtotal**		**730 (43.7)**	**100 (13.7)**	**52 (7.1)**	**213 (29.2)**	**14 (1.9)**	**351 (48.1)**

M = male

In Study 1, *Ph*. *neglectus* was found to be the prevalent species in all districts monitored (95.6% among all phlebotomines; 98.9% among *Larroussius* species). It was collected into and around sites close to recent VL cases, especially near chicken coops and animal pens. The other *Larroussius* species identified were *Ph*. *tobbi* (0.9%) and *Ph*. *perfiliewi* (0.2%). A few specimens of *S*. *minuta* (18) were also recorded.

In Study 2, only 35/91 sites monitored (38.5%) were positive for sand flies: 15/20 (75.0%) in Vlorë, 23/30 (76.7%) in Gjirokastër and 7/41 (17.1%) in Korçë. The majority of sand fly specimens (425) were from Vlorë country (58.2%), particularly from Sarandë and Vlorë districts. In this study the prevalent species was found to be *Ph*. *perfiliewi* (29.9% among all phlebotomines; 56.2% among *Larroussius* species), whereas *Ph*. *neglectus* and *Ph*. *tobbi* accounted for 13.7% and 7.1% of specimens, respectively. *Ph*. *similis* (1.9%) was only recorded in three close sites of Dropull, Gjirokastër district. The highest elevation of a positive site was at 1,185 m a.s.l., where one *Ph*. *neglectus* and one *S*. *minuta* were caught. The latter species represented 48.1% of all specimens collected in this study.

In Study 3, *Ph*. *neglectus* was the most prevalent species (55.8%) followed by *Ph*. *tobbi* (37.5%). The other two species identified, not associated to VL transmission, were *Ph*. *papatasi* (1.3%) and *S*. *minuta* (5.4%).

### Detection of *Leishmania* infections in sand flies

**Fig 3:**
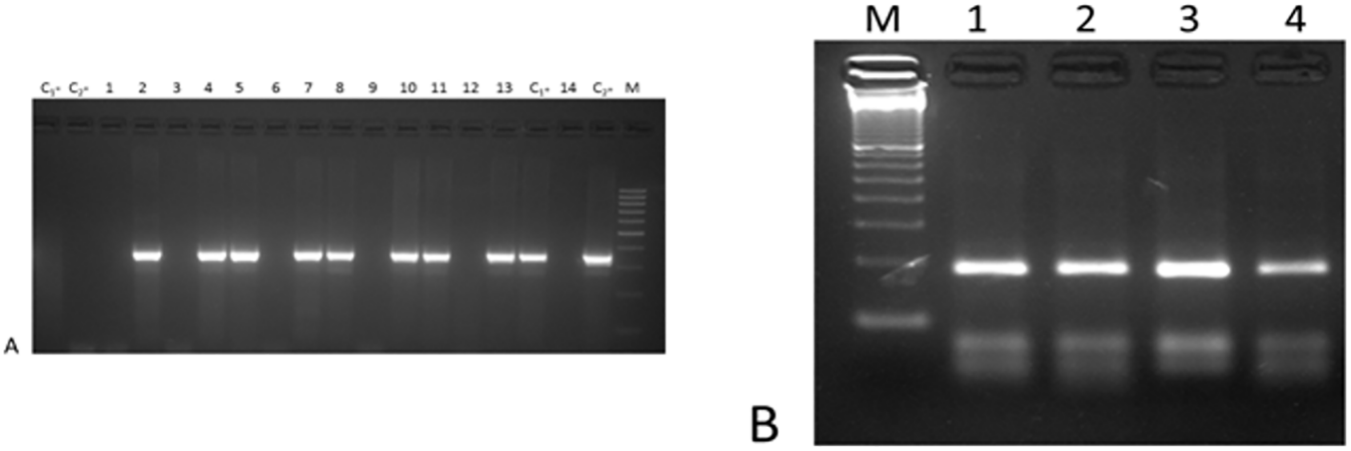
Molecular detection and characterization of *Leishmania* from wild-caught sand flies in Albania. **(A)** Nested-PCR targeting a *Leishmania* sp. small-subunit ribosomal DNA sequence. Lane C_1_-: uninfected reared sand fly DNA; lane C_2_-: PCR Master Mix with no DNA; lanes 2, 4, 5, 7, 8, 10, 11: positive *P*. *neglectus* pools; lane 13: positive *P*. *tobbi* pool; lanes 1, 3, 6, 9, 12, 14: negative *P*. *perfiliewi* pools; lane C_1_+: *L*. *infantum* promastigotes DNA; C_2_+: *L*. *infantum* promastigotes DNA mixed with uninfected reared sand fly DNA; lane M: 100 base pair ladder (Promega). **(B)** ITS-1 n-PCR-RFLP for *Leishmania* species characterization. Lane M: 100 base pair ladder (Promega); lane 1: *Leishmania* isolate from Albanian *P*. *neglectus* (IMJN/AL/2011/MJN2-ISS3056); lane 2: *L*. *infantum* (human isolate from Albania, MHOM/AL/2006/ISS2840); lane 3: *L*. *infantum* (dog isolate from Albania, MCAN/AL/2006/ISS2841); lane 4: WHO reference strain for *L*. *infantum* (MHOM/TN/1980/IPT-1).

In Study 1, n-PCR was used to detect genomic *Leishmania* DNA in 425 *Phlebotomus* (*Larroussius*) females after species identification. The average number of specimens per pool was 8 (range: 3–12). In total, 8/54 pools (14.8%) were found positive for leishmanial DNA, of which 7/47 pools made from 422 *Ph*. *neglectus*, and one pool made from *Ph*. *tobbi*, consisting of the only 3 specimens collected in Permet district (Fig 3A).

Regarding the spatial distribution of *Ph*. *neglectus*-positive pools, 2 were from Kolonje, 1 from Kruje, and 4 from Lezhë district, of which 3 from the Lezhë suburban VL focus of Koder-Marlekay. None of the 7 *Ph*. *perfiliewi* pools collected from Vlorë and Sarandë districts (a total of 44 specimens) was found positive for leishmanial DNA.

In Study 3, two of 387 sand fly females dissected were found to harbour promastigotes (0.9%). Table 5 reports the number of females examined and the species identified for each site.

**Table 5:** *Leishmania* infections detected in wild-caught sand flies in sites of Lezhë district, September 2011 (Study 3).

Site	Altitudes (m a.s.l.)	VL cases recorded **	No. of flies dissected	Species (no. of *Leishmania* positive; percentage)			
				*P*. *neglectus*	*P*. *tobbi*	*P*. *papatasi*	*S*. *minuta*
Kodër Marlekaj*	43	2	69	64 (2; 3.1%)	5 (0)	0	0
Tresh	96	2	308	147 (0)	138 (0)	5 (0)	18 (0)
Grykë Manati	31	1	7	2 (0)	2 (0)	0	3 (0)
Manati	26	1	3	3 (0)	0	0	0
**Total**		**6**	**387**	**216 (2; 0.9%)**	**145 (0)**	**5 (0)**	**21(0)**

(*) This site is a peri-urban settlement of Lezhë town.

(**) In the previous two years

The two positive specimens were from a sample of 64 *Ph*. *neglectus* collected in Koder-Marlekaj (3.1%). Interestingly, a similar n-PCR positive rate (3.5%) was estimated in a pool of this species collected in this site in 2006 (see above). One specimen had a scanty infection with few parasites attached to the wall of the stomodeal valve. The other fly harboured a massive mature infection, with a abundant metacyclic promastigotes. (S1 Video). Parasites have been successfully cultured only from this specimen. The strain (IMJN/AL/2011/MJN2-ISS3056) was identified by as belonging to *L*. *infantum*. ITS-1 n-PCR-RFLP showed the specific pattern of this species (184-72-55 bp bands) (Fig 3B), whereas MLEE identified the strain as belonging to zymodeme MON-1.

## Discussion

Both VL and CL caused by *L*. *infantum* are emerging diseases in Albania. In 2003, Velo et al [15] reported an increasing trend of VL cases, from 144 to 209, occurred between 1997 and 2001. More recently, Petrela et al [2] have confirmed this trend in a retrospective analysis of data recorded from 1995 to 2009 at the national pediatric reference hospital of Tirana. This analysis showed that VL is largely a paediatric disease, with an incidence rate of 25/100,000 in the age group 0–6 years for that hospital area which is much higher than in other endemic countries of southern Europe [1]. A most recent gap analysis from 2005–2013 shows a decreasing trend of VL from 2.5/100000 to 0.8/100000. Dogs are the only confirmed reservoir of zoonotic VL in Albania. As a result of uncontrolled increase in number of stray and owned dogs, canine leishmaniasis is being increasingly observed in several Albanian districts although systematic monitoring of the disease in dogs has not been performed yet. Serosurveys performed on some 400 dogs from 7 districts disclosed an IFAT rate of 15.8% [16].

Overall, our studies indicate that *Ph*. *neglectus* is the dominant *Larroussius* species associated to foci of VL in Albania. This species, also referred as to *Ph*. *major neglectus* [17], is a sand fly member of the *Ph*. *major* complex [18], which has long been suspected to be involved in the transmission of VL in eastern Mediterranean countries [19,20]. In 1988 Léger et al [6] firstly reported from the island of Corfu (Greece) the natural infection of this species with *L*. *infantum* zymodeme MON-1. One year later, Garifallou et al. [20] reported on the natural infection by *L*. *donovani sensu lato* (most probably *L*. *infantum*) of a *Ph*. *neglectus* specimen from the island of Zakinthos (Greece). Ivović et al [21] found promastigotes in dissected females of this species collected in the Bar area of Montenegro, but parasites were not identified. Taking into account morphological characters that distinguish *Ph*. *neglectus* from other members of the *Ph*. *major* group [22], the geographical distribution of this species appears to be restricted to the eastern Mediterranean countries of Europe and western border of Middle East. Besides Albania, this species was recorded in Bulgaria [23], Cyprus [24], Croatia [25], Greece [26], Hungary [27], Kosovo [28], Italy [29], Malta [30], Montenegro [21], Romania [31], Serbia [32], Slovenia [29], and Turkey [33].

The biology of *Ph*. *neglectus* in Albania needs to be investigated further; however some data on seasonal dynamics and host preferences have been recorded from 6 collecting sites of Kruje and Lezhe districts in 2002. First adults were collected by CDC traps on June 11^th^ and the last ones on October 16^th^. The highest number of specimens was recorded at the end of July. Interestingly, blood-meal analysis from about fifty blood-fed specimens collected, showed that among 5 *Phlebotomus* species examined, *Ph*. *neglectus* was the only species found with human (6%) and canine blood (17%) [7].

Despite a pool made from 3 specimens of *Ph*. *tobbi* collected in the southernmost part of the country tested *Leishmania* positive by a molecular method in Study 1 in 2006, we failed to demonstrate microscopically the occurrence of natural infections in 145 *Ph*. *tobbi* specimens examined in north Albania in Study 3. In general, *Ph*. *tobbi* was found low prevalent in several Albanian sites associated to VL transmission as compared with *Ph*. *neglectus* (see Table 2). However this species may play the role of a secondary vector in Albania, considering that *Ph*. *tobbi* has been incriminated as *L*. *infantum* vector in Cyprus [6], in western and eastern parts of Turkey [34,35], and it is suspected to have such a role in neighbouring countries such as Croatia and Greece [36].

*Ph*. *perfiliewi* was found abundant only in catches performed using UV light-Onderstepoort-type traps placed near farms housing large livestock, and in sites selected for purposes unrelated to VL transmission. Conversely, the relative prevalence of this species was almost nul in sites targeted because of recent occurrence of VL cases. Furthermore, no *Leishmania*-PCR positive specimens were recorded in 7 pools from this species. We cannot exclude that the use of a trap developed for *Culicoides* collection may have provided a biased species prevalence of the sand fly fauna.

The *Leishmania* typing methods employed by us either require a sufficient amount of intact DNA (for PCR-RFLP), or parasite isolation and mass cultivation (for MLEE). DNA material left from the eight nPCR-*Leishmania* positive pools proved insufficient, or possibly degraded, for the PCR-RFLP amplification protocol. Furthermore, one of the two infected *P*. *neglectus* specimens had a scanty infection of promastigotes which did not grow in culture and were lost for molecular typing.

Altogether our studies reasonably indicate that *Ph*. *neglectus* is the main vector of human leishmaniasis in Albania, as three main vector incrimination criteria (recently reviewed in [36]) were satisfied: i) *Ph*. *neglectus* is associated with biotopes of human VL transmission; ii) it exhibits anthropophily and also feeds on the canine reservoir host; iii) this species was found to harbour infectious stages of the *Leishmania* species and zymodeme causing human and canine disease in Albania.

## Supporting information

S1 Video *Leishmania infantum* promastigotes present in dissected wild-caught *Ph*. *neglectus* in sites of Lezhë district, September 2011 (Study 3). (WMV)
